# Scent detection dogs detect a species of hard tick, *Dermacentor albipictus*, with comparable accuracy and efficiency to traditional tick drag surveys

**DOI:** 10.1186/s13071-024-06519-8

**Published:** 2025-04-02

**Authors:** Troy Koser, Aimee Hurt, Laura Thompson, Alyson Courtemanch, Benjamin Wise, Paul Cross

**Affiliations:** 1 Montana State University, Montana, USA; 2 Working Dogs for Conservation, Montana, USA; 3 National Climate Adaptation Science Center, U.S. Geological Survey, Knoxville, TN USA; 4 Wyoming Game and Fish Department, Wyoming, USA; 5 Northern Rocky Mountain Science Center, U.S. Geological Survey, Montana, USA

**Keywords:** *Alces alces*, *Dermacentor albipictus*, Greater yellowstone ecosystem, Hard ticks, Moose, Scent detection dogs, Surveillance, Tick drag, Winter ticks

## Abstract

**Background:**

Accurate surveillance data are critical for addressing tick and tick-borne pathogen risk to human and animal health. Current surveillance methods for detecting invading or expanding tick species are limited in their ability to scale efficiently to state or national levels. In this study we explored the potential use of scent detection dogs to assist field surveys for a hard tick species: *Dermacentor albipictus*.

**Methods:**

We used a series of indoor and in situ training simulations to teach scent detection dogs to recognize *D. albipictus* scent, distinguish tick scent from associated vegetation, and develop a cautious search pattern. After training, we deployed both a scent detection dog survey team and a human-only survey team on transect and surveillance plot surveys then compared the detection rates and efficiency of both methods.

**Results:**

Scent detection dogs required more time and money to train on field surveys but were comparable to traditional tick drags when accounting for cost per unit area surveyed. There was a lack of agreement on positive (ticks present) versus negative (ticks not present) sites between the two methods, implying that neither method is particularly reliable at detecting *D. albipictus*.

**Conclusions:**

Estimating detection bias and false negative rates for tick surveillance methods such as tick drags will be important for accurately evaluating tick-borne disease risk across space and into the future. We found scent detection dogs to be a reasonable alternative sampling approach to consider when ticks are at low abundance or patchily distributed such as during tick range expansion or novel invasions. Scent detection dogs may also be useful for sampling for ticks in areas or along surfaces that are difficult to sample with the traditional tick drag technique like at ports of entry or livestock competitions.

**Graphical Abstract:**

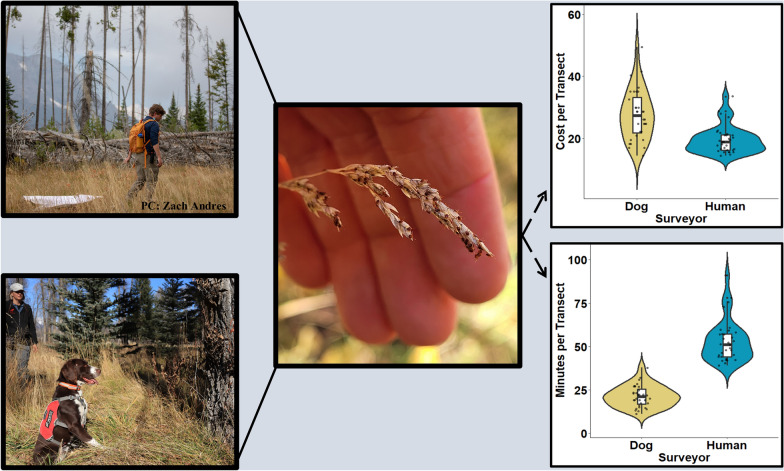

## Background

Ticks, specifically ixodid (hard) tick species, pose a significant and growing threat to human and animal health in the USA and around the world. Ticks are responsible for vectoring pathogens causing > 90% of nationally notifiable human vector-borne disease cases reported in the USA and are major pests in the cattle industry and some wildlife conservation settings [1–5]. The recognition of several novel human and animal pathogens in ticks and associated hosts, as well as a wide diversity of potentially pathogenic agents, has increased awareness to the emergence of tick-borne disease threats in the USA [6–8]. Furthermore, climate change has been linked to longer active seasons and increased reproduction rates in important tick vectors like *Ixodes scapularis* and, alongside human movement of ticks and their hosts, creates new opportunities for species invasion or range shifts [9–12].

Understanding current trends in tick distribution and abundance is critical to estimating tick-borne disease risk and investigating the interactions between ticks, hosts, and the environment. A common technique for surveying tick populations is in situ or active surveillance for ticks seeking a host (*i.e.*, “questing”) through techniques like dragging a flannel cloth across a prescribed survey path which provides information on tick species presence and density [13, 14]. Pathogen prevalence of infected ticks can be determined from drag surveys if collected ticks are tested for specific disease-causing agents. Dragging for ticks involves relatively low resource investment and has similar detection rates when compared to other methods of tick collection like dry ice-baited traps [15–18]. Though tick drags are by far the most common tick population survey technique, survey effectiveness depends on the spatial scales, tick species attachment behavior(s), and habitat types or other environmental conditions under investigation [15, 17]. For surveys aimed at detecting rare or less conspicuous tick species, as may be the case for an invading or expanding species, techniques for sampling larger areas or with greater detection probabilities may be necessary.

Scent detection dogs have been used to detect several inconspicuous, cryptic, or rare wildlife species including koala (*Phascolarctos cinereus*), Franklin’s ground squirrel (*Poliocitellus franklinii*), and brown marmorated stink bugs (*Halyomorpha halys*) [19–21]. Though resources are needed to train scent detection dogs to find targets in field contexts without destroying or harming remains or animals, survey time per area is often shorter and detection rates higher than other methods [22–25]. For example, overall detection rates and probability of detection (given presence) for black bears (*Ursus americanus*), fishers (*Martes pennanti*), and bobcats (*Lynx rufus*) during scat surveys conducted with scent detection dogs were higher when compared to hair snare and camera surveys [26]. Scent detection dogs may prove a valuable tool in determining tick presence and abundance at previously unfeasible scales and study contexts such as widespread invasive tick surveillance given the variable efficacy of existing detection methods like dragging surveys.

In this study we experimentally trained scent detection dogs to recognize the target scent of a hard tick species, the winter tick (*Dermacentor albipictus*), and conducted field trials to compare accuracy and efficiency to human-conducted surveys. The winter tick is a widespread one-host tick in North America which attaches to a wide range of hosts but reaches high infestation loads on large ungulates like moose (*Alces alces*), elk (*Cervus canadensis*), and caribou (*Rangifer tardus*) [42]. *Dermacentor albipictus* are known for infesting moose at densities high enough to lead to anemia and even death, with winter tick epizootics being linked to moose population declines at the southern end of their range [5, 27–29]. Tracking *D. albipictus* expansion into the northern reaches of Canada and southern Alaska is of concern for ungulate population managers [30–32]. Here we present the first documented attempt, to our knowledge, to train and deploy scent detection dogs to survey for a hard tick species, *D. albipictus*, in situ. We also provide background on training scent detection dogs to identify tick scent and teaching an appropriate search pattern. Finally, we compared the performance and resource investment of a scent detection dog-assisted survey team to a traditional tick drag survey.

## Methods

### Preliminary training

In 2020 two scent detection dogs with Working Dogs for Conservation (WD4C), an adult female Belgian Malinois (Tule) and an adult female Labrador retriever (Lily), began training exercises at the WD4C facility in Turah, Montana to determine their ability to recognize *D. albipictus* scent using wild-caught winter tick larvae. Both dogs demonstrated some ability to recognize *D. albipictus* scent in controlled, indoor settings when trained to identify PVC elbow joints containing ticks versus control joints. Both dogs could also identify containers with ticks present and distinguish from control containers on an outdoor trail. These findings warranted further exploration of their ability to detect questing ticks in the field in Jackson Hole, Wyoming. Lily retired at the end of 2020, thus field training continued with Tule and Frost, an adult male springer spaniel mix. Both Tule and Frost were selected to carry out field training for winter tick surveys because both dogs had experience in relevant field studies. Tule was already exposed to tick scent during preliminary training and feasibility testing in Turah, Montana while Frost had previous experience with hand presentations and searching for targets at head height where clusters of tick larvae were most likely to be present.

### Field training

In 2021 Tule and Frost began target scent recognition training and field exercises for *D. albipictus* surveys with their handler in Jackson Hole, Wyoming. The valley of Jackson Hole is situated in northwestern Wyoming, south of Yellowstone National Park and contains Grand Teton National Park, Bridger-Teton National Forest, the National Elk Refuge, and the residential areas surrounding the towns of Jackson and Wilson. Elevations range from ~1850 m in the Snake River flood plain to ~4200 m in the Teton Range. Vegetation at lower elevations includes sagebrush (*Artemisia* spp.) communities with willow (*Salex* spp.) and cottonwood (*Populus angustifolia*) galleries in riparian areas. Mixed conifer and aspen forests are present at mid-elevations including lodgepole pine (*Pinus contorta*), Douglas fir (*Pseudotsuga menziesii*), and aspen (*Populus tremuloides*). At mid-high elevations, spruce (*Picea engelmannii*) and subalpine fir (*Abies lasiocarpa*) are common.

**Fig. 1:**
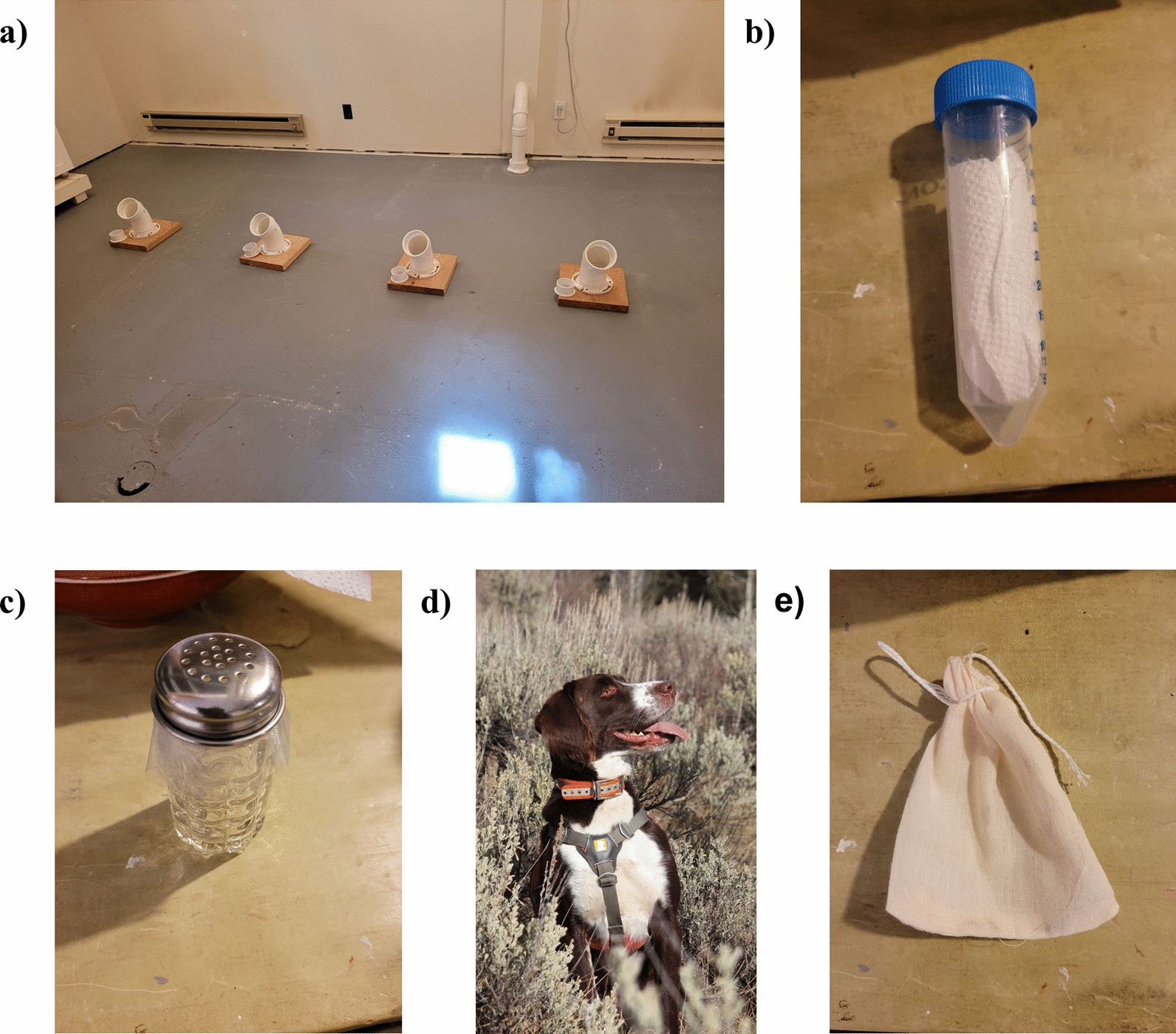
**a** PVC elbow array used in indoor training of scent detection dogs to recognize *Dermacentor albipictus* scent and distinguish from associated vegetation. **b** The 50 ml Falcon^®^ tube with paper towel and holes drilled on top to allow airflow, covered in organza. **c** Salt and pepper shaker with organza cloth covering holes. **d** Frost, a male springer spaniel mix at a survey site. **e** A muslin bag

During initial scent-imprinting, dogs were “marked” by the handler with an auditory cue when they smelled target scent and rewarded with either food or toy play. After imprinting of tick scent and dogs showed scent recognition, the dogs were expected to perform a trained final response (TFR), a previously trained behavior which was a sit with either a point at the handler or in the direction of the target scent when they pinpointed the source of target scent. Indoor training used a PVC elbow array where ticks with associated vegetation and three control containers with vegetation were presented to the dogs in various containment systems designed to keep ticks contained but allow air and scent cue to flow (Fig. 1). “Known” trials allowed the handler previous knowledge on elbow contents while "blind" trials only allowed the recorder to know elbow contents. During blind trials the handler and dog would stay in a separate room while the recorder organized the trial using gloves to prevent contaminating scent profiles. Once prepared, the recorder would signal to the handler that the trial could commence and record every instance of alert or changes in behavior (CoB) as dictated by the handler. Containment systems included salt and pepper shakers with openings covered by organza, Falcon® tubes with organza coverings, and muslin bags (Fig. 1). Falcon tubes and salt and pepper shakers with organza coverings produced the most reliable results while also preventing ticks from escaping the PVC elbows.

**Fig. 2:**
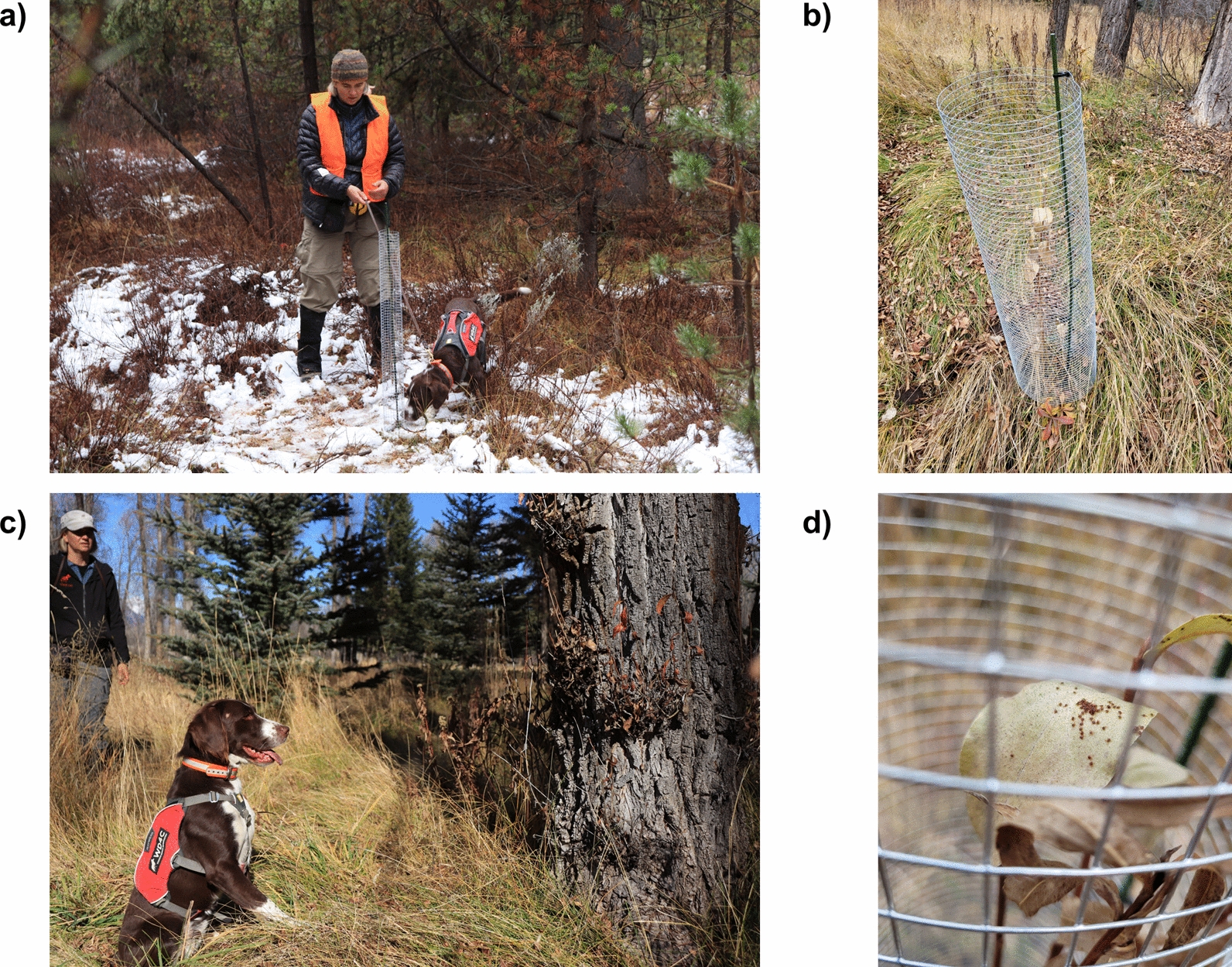
**a** Frost, a male springer spaniel mix, checking a wire mesh cage with handler, Aimee Hurt, during *Dermacentor albipictus* field survey training exercises. **b** Wire mesh cage erected around a known cluster of questing *D. albipictus* larvae used in field training exercises. **c** Frost performing a trained final response (TFR) during field training. **d** Questing larval *D. albipictus* on vegetation within a wire mesh cage

The primary difficulty in progression to in situ tick detection was training a search pattern where dogs would carefully search vegetation tips for questing larval clusters without destroying clusters so known clusters could be used for repeated trials. We used a variety of mechanisms surrounding known clusters of questing larval ticks to prevent disturbance by the dogs while searching and to allow for repeated trials, including wrapping vegetation tips in large organza bags and placing 1-cm wire cages around vegetation (Fig. 2). Wire mesh cages around known tick clusters proved the best equipment for training target scent context and allowed for repeated trials. Indoor and in situ field training required 6 working days where dogs were actively trained on scent recognition and search patterns. Training also included 2 rest days where dogs were given time to process their training and technicians could assemble new training courses. The final 2 days of field training included twelve blind trials where scent detection dog and handler were presented a series of four “hot” cages with tick clusters and four negative control cages along a course. One dog, the Springer spaniel mix named Frost, established an effective search pattern and demonstrated the ability to reliably detect known tick clusters and reject controls using training and validation experiments similar to those reported in other scent detection dog studies [23–25].

### Field surveys

We deployed both the scent detection dog-assisted team (Frost, handler, survey guide) and a human-only traditional tick drag team on field surveys for *D. albipictus* over the course of 2 days. Each survey location was first sampled by the scent detection dog-assisted team to prevent scent contamination and bias from human-only surveys. A technician without knowledge of the dog-assisted survey would then conduct a traditional human-only drag survey, followed finally by a guide from the scent detection dog surveys who dragged or flagged locations marked by the dog-assisted team. Marked locations from the dog-assisted survey were sampled after the traditional drag survey to avoid potential bias in technician drag patterns if they were to notice evidence of previous activity like bent vegetation or flattened grass. Two forms of field trials were undertaken: 250 m transects and 500 m × 500 m surveillance plot surveys. The 250 m transect surveys are representative of a typical tick abundance and environmental association study where transects are stratified across environmental conditions of interest then sampled [13–15]. We stratified 32 transects in suitable *D. albipictus* habitats across the Jackson Hole valley in cottonwood/riparian, sagebrush/mixed aspen, mixed forb/grass, and conifer-dominated habitats at low (~ 1870 m), medium (~ 1980 m), medium-high (~ 2100 m), and high elevations (~ 2200 m) and allowed the scent detection dog and handler to search with a guide to ensure searching was within ~ 10 m of the transect GPS path. Once the scent detection dog either performed a TFR or demonstrated “change of behavior” (CoB), the handler would indicate to the guide which individual plant or cluster of vegetation was likely targeted. The guide would then record a GPS point and take a picture of the site for sampling and recorded the distance from the nearest point on the transect to the indicated vegetation. A change of behavior was called by the handler when the dog changed from standard searching behavior to a set of innate behaviors consistently expressed in the presence of target odor, including a more excited state with exaggerated tail wagging and increased sniffing rate or intensity. After the scent detection dog team completed surveys, a technician with no knowledge of the dog-assisted survey detections would sample the same transects using a traditional human-only drag method. Dragging involved moving a 1 m^2^ flannel cloth across available vegetation while tracking progress using a GPS device. The surveyor would check the drag cloth for ticks roughly every ten paces and remove any attached ticks using a lint roller. Ticks were later quantified and ~10 ticks per detection event were preserved in 75% ethanol to confirm species identity using a dichotomous key [33]. Upon tick detection, the surveyor would retrace their path to find the most likely origin source of ticks and record GPS location, vegetation type, and vegetation height. To ensure all ticks from the identified cluster were collected, the surveyor would then conduct a 10 m radius drag by dragging in a spiral away from the focal detection point. All ticks collected during origin-tracing and radius drags were given a “detection event” label. Once the human surveyor sampled a transect, the guide from the scent detection-assisted surveys then sampled TFR and CoB locations using a 10 m radius drag. None of the radius drags from scent detection dog-assisted surveys overlapped within 5 m of a human surveyor detection event, but if overlap were to occur, we would count the total tick abundance from an overlapping detection event toward both survey types.

Surveillance plots were designed to simulate a survey type more suited to detect a rare or invading tick species across a large area. We outlined three 500 m × 500 m surveillance plots in the northern, middle, and southern portions of Jackson Hole using flagging tape and flags and set a 500 m search path within the plot. As above, surveillance plots were first sampled by the scent detection team then a human surveyor and finally the guide from the scent detection team.

Transect results were analyzed using a McNemar’s *χ*^2^ test from the *stats* package in R and larvae per cluster, larval density, and larval abundance per transect results were analyzed using a generalized linear model with a “*quasipoisson*” distribution in the package *lme4* [34, 35].

The time required to complete transects and surveillance plots was recorded for both methods. Overall costs for training and per transect and surveillance plot were calculated using the 2021 General Schedule (GS) pay rate for a GS-9 Step 1 field technician at $22.08/h rate for the human-only survey team and $73.75/hour for the dog handler salary, which includes insurance and care for the scent detection dogs (Table 2).

## Results

### Indoor and field training

One scent detection dog, Frost, demonstrated the ability to successfully identify *D. albipictus* larval cluster scent [known trials (*n* = 2) and blind trials (*n* = 4): 100% positive identification rate] and reject control or vegetation-only containers [known trials (*n* = 2): 100% rejection rate for known trials, blind trials (*n* = 4): 80% rejection rate] in controlled indoor trials. Frost was also able to correctly identify all cages containing tick clusters and reject controls in 11/12 field training trials and established a careful search pattern where Frost would search tips of vegetation for tick clusters with minimal disturbance. Field training, including dog handler salary, dog insurance and health care, supplies, lodging, and per diem, required 48 active working hours and cost $4246. Training for a human tick drag technician required 8 working hours and cost $376.64 including salary, supplies, and lodging (Table 2).

### Field surveys

Field surveys were designed to compare a typical *D. albipictus* survey using the tick drag method to a survey by a scent detection dog team. All ticks preserved in ethanol from both survey types were confirmed as *D. albipictus* larvae using a dichotomous key. Ticks were detected on 11 of 32 (34%) transects and in all three surveillance plots surveyed by the scent detection dog team while the traditional tick drag method found ticks on 14 of 32 (44%) transects and in all three surveillance plots. Six transects were found positive for ticks using both methods leaving 13 discordant pairs where dog-assisted and human-only tick drag surveys did not agree on positive versus negative status. Results from the McNemar’s *χ*^2^ test did not reveal considerable differences between the predictive accuracies of the two survey methods (Table 1, *χ*^2^ = 0.308, *df* = 31, *P* = 0.579).

**Table 1:** *Dermacentor albipictus* larvae detection results for two survey types: scent detection dog-assisted and human-only traditional tick drag surveys. Both teams surveyed the same 32 250 m transects stratified across Jackson Hole, Wyoming

Scent detection dog surveys	Human-only surveys	
	Positive	Negative
Positive	6	5
Negative	8	13

**Fig. 3:**
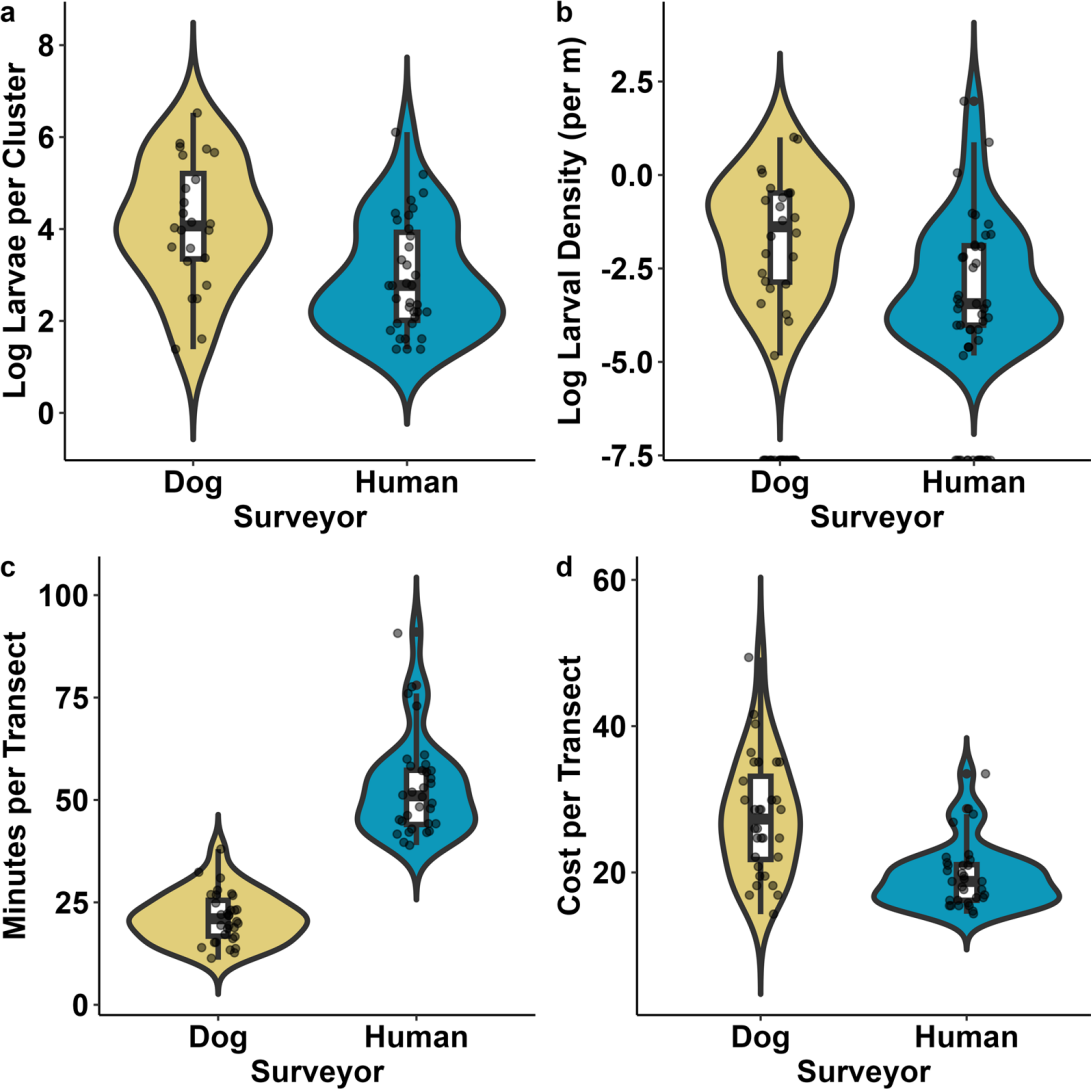
**a** Violin and boxplots visualizing the number of *Dermacentor albipictus* larvae detected per cluster identified in surveillance plots and transects, on a logarithmic scale, surveyed by human-only team using traditional tick drag method and a team assisted by a scent detection dog with handler. **b** Larval density detected via human-only tick drags versus dog-assisted teams on a logarithmic scale. **c** Visualizations of minutes spent to survey 32 individual 250 m transects by both human-only and scent detection dog-assisted teams. **d** Visualizations of dollars spent to survey 32 individual 250 m transects based on $22.08/h rate for a single human surveyor and $73.75/h for scent detection dog-assisted team plus $22.08/h rate to survey Trained Final Response (TFR) locations

We found 66% fewer *D. albipictus* larvae in larval clusters in the human-only tick drag surveys [mean = 45, 95% confidence interval (CI) = 17–72] compared with dog-assisted surveys (mean = 132, 95% CI = 63–201, *t*_(106)_ = −2.70, *P* = 0.009, Fig. 3a). We observed a small difference of 6% fewer larvae per meter surveyed on human-only tick drag surveys compared to dog-assisted surveys (mean human = 0.25 larvae per meter, 95% CI = 0–0.54, mean dog = 0.24 larvae per meter, 95% CI = 0.08–0.39, *t*_(34)_ = 0.088, *P* = 0.933, Fig. 3b). The scent detection dog-assisted surveys detected a total of 17 larval clusters on 32 transects and 21 clusters on 3 surveillance plots while the traditional tick drag method detected 29 clusters on transects and 21 clusters on surveillance plots.

Ticks were detected on 31 of 50 total (62%) TFRs and CoBs called by a scent detection dog and their handler across both transect and surveillance surveys. A total of 23 out of 28 (82%) TFRs were positive for ticks with an average cluster size of 123 larvae per detection (95% CI = 61–184) while 8 out of 22 (41%) CoBs were positive for ticks and on average yielded 24 larvae per detection (95% CI = 0–57). In total, 13 out of 25 (52%) of all TFRs and CoBs were positive for ticks on the first day of field surveys while 18 of 25 (80%) were positive on the second day.

On average, both the scent detection dog team and traditional tick drag methods found a larval cluster every 5 m. The scent detection dog and handler also identified four tick clusters off-transect while on-route to survey sites. Distance from transect or search route line to identified tick cluster for the scent detection dog team was on average 109 cm for TFRs (95% CI = 56–161) and 93 cm for CoBs (95% CI = 4–183).

The average time to complete a 250 m transect for the scent detection dog, handler, and tick drag technician was 24 min (95% CI = 21–27) costing $27 per transect (95% CI = $24–$30) when accounting for salary alone compared to 53 min (95% CI = 49–58) and $20 per transect (95% CI = $18–$21) for the traditional tick drag method using a single tick drag technician (Figs. 3c and d). The average time to complete a 500 m × 500 m surveillance plot for the dog-assisted team was 122 min (95% CI = 107–137) and cost $123 per plot (95% CI = $115–$131) compared with 144 min (95% CI = 93–194) and $53 per plot (95% CI = $34–$72) for the traditional drag method. The combined efforts of dog handler, dog, and tick drag technician took 16 h or 2 working days to survey 32 250 m transects and cost a total of $1275.32 ($39.84 per transect), including salary, per diem, and lodging compared with 29 h or 4 working days and $840.32 ($26.25 per transect) for a tick drag technician on their own (Table 2). The dog-assisted team required 7 h or 1 day to survey three 500 m × 500 m surveillance plots costing $711.41 ($237.14 per plot) while the traditional tick drag method took 8 h or 1 day and cost $226.64 ($75.55 per plot).

**Table 2:** Estimated costs for training a scent detection dog team to find a hard tick species in the field compared to a human-only tick drag survey. We also compared costs and hours/days worked to survey 32 250 m transects stratified across Jackson Hole, Wyoming and three 500 m × 500 m surveillance plots

Type of cost	Survey method	Costs	Cost per unit	Units	Total cost (USD$)
Training	Human-only tick drags	Training in WY	$22.08/h	8 h	176.64
		Supplies	Drag cloth, gear, etc.		150
		Lodging	$50 per day	1 day	50
		Total		1 day	376.64
	Dog team	Training in Wyo^a^	$73.75/h	48 h	3540
		Supplies	PVC elbows, etc.		100
		Handler per diem	$51 per day	6 days	306
		Lodging	$50 per day	6 days	300
		Total		8 days^b^	4246
32 Transect surveys	Human-only tick drags	Salary	$22.08/h	29 h	640.32
		Lodging	$50 per day	4 days	200
		Total		4 days	840.32
	Dog team	Handler service fee^a^	$73.75/h	12 h	885
		Handler per diem	$51 per day	2 days	102
		Technician salary	$22.08/h	4 h	88.32
		Lodging (handler)	$50 per day	2 days	100
		Lodging (technician)	$50 per day	2 days	100
		Total		2 days	1275.32
3 Surveillance plot surveys	Human-only tick drags	Salary	$22.08/h	8 h	176.64
		Lodging	$50 per day	1 day	50
		Total		1 day	226.64
	Dog team	Handler service fee^a^	$73.75/h	7 h	516.25
		Handler per diem	$51 per day	1 day	51
		Technician salary	$22.08/h	2 h	44.16
		Lodging (handler)	$50 per day	1 day	50
		Lodging (technician)	$50 per day	1 day	50
		Total		1 day	711.41

^a^Handler service fee includes handler salary, dog care, insurance, and food as well as operational costs for WD4C

^b^Scent detection dogs require 2 days of rest for every 5 days of work

Overall survey speed for the scent detection team across both survey types was 11 m/min (95% CI = 10–13) and 5 m/min (95% CI = 4–6) for the human-only tick drag method.

## Discussion

These field trials represent the first documented attempt to train and deploy scent detection dogs to search for a hard tick species in the field. Winter tick larval cluster sizes detected by a scent detection dog tended to be larger than those detected via human-only dragging, implying that scent strength may relate to cluster size (Fig. 3a). Finding larger clusters of ticks may be desirable for risk-assessment purposes in tick-borne disease systems where infestation burdens are relevant for pathology as is the case for the *D. albipictus*-moose and cattle fever tick systems. Though the number of tick-positive transects and number of detection events overall did not differ remarkably between dog-assisted and human surveys (Table 1), dog-assisted surveys were roughly two times faster, which may prove critical for surveillance projects spanning large areas (Fig. 3c). A scent detection dog team costs roughly three times as much as a human-only surveyor on an hourly basis and required site revisits to sample TFRs and CoBs, limiting the cost-effectiveness of dog surveys compared with the traditional tick drag method for the 32 transects and 3 surveillance plots examined in this study (Fig. 3d). Field training and teaching appropriate search patterns required 8 days and $4246 in direct costs for the scent detection dog team, which was more than ten times the training costs for a human surveyor at $376.64 in direct costs and one training day. We expect field training time and costs for the scent detection dog team to decrease in subsequent years of tick sampling since dogs have shown the ability to recognize target scents for long periods of time [23–26]. Interestingly, we found no overlap (within 5 m) in detection events between a scent detection dog-assisted survey team and the traditional tick drag method, implying that both methods have imperfect detection probabilities and highlighting the need for repeated sampling design, additional exploration of alternative survey methods, and accounting for detection probabilities in the analysis of tick abundance data.

While larvae were not recovered in 38% of overall alerts (both full TFRs and CoBs) made by the scent detection dog team, it is possible that questing larvae were present but not found via the imperfect drag method. Additionally, false positive rates changed over time potentially as both dog and handler learned the context of target scent cues and as Frost was able to be rewarded immediately for performing a TFR on visible tick clusters. Except for a few high infestation cases of thousands of larvae on the tips of vegetation, larval *D. albipictus* clusters are difficult to immediately identify and, thus, difficult to quickly reward Frost for positive identification. Even so, a handful (*n* = 8) TFRs or CoBs were able to be immediately identified as positive for tick larvae without dragging, which may have reinforced field training and led to decreasing false positive rates over time. We believe that it is likely that Frost’s detection performance would have continued to improve with additional time and financial resources for training.

Determining tick species distribution and abundance at larger scales is vital to accurately assessing current and future disease risk. A major hindrance to widespread active surveillance for ticks has been logistic constraints; unlike other vectors such as mosquitoes, which can be caught with traps near urban centers, only a handful of tick vectors are effectively trapped with dry ice traps and host-trapping grids are not easy to monitor by groups such as pest management councils [18, 36, 37]. Here we explore a potential efficient tick surveillance method using scent detection dogs, a growing resource for conservation and other groups around the USA. Future research could investigate the ability of dogs to differentiate between species of ticks, which seems plausible given their ability to discriminate between the sign of closely related species such as grizzly (*Ursus arctos*) and black (*Ursus americanus*) bear scat [38]. Such an ability would be especially useful if dogs were to be used to search ports-of-entry, international livestock shows, or other potential introduction avenues for high-risk tick species. Tick systems like the cattle fever tick (*Rhipicephalus microplus*) system on the USA–Mexico border may benefit from increased survey speeds and the ability to detect ticks on hosts at potential invasion hot spots [39]. The invasion and spread of the long-horned tick (*Haemaphysalis longicornis*) across the eastern USA is another example of a system where widespread surveillance for questing ticks and attachment to livestock hosts may benefit from faster methods [40, 41].

## Conclusions

Scent detection dogs may benefit widespread, well-funded tick surveillance projects, but the traditional tick drag method is still likely to be the most cost-efficient tick surveillance approach for ecological association or abundance surveys. The lack of agreement between tick status using both methods implies important limitations in detection probability and accuracy for the widely used tick drag method and raises the need to account for detection biases and estimating false negative rates if tick drag data are used to extrapolate distribution or abundance across out-of-study areas [14, 15, 17]. Overall, we found that dog-assisted crews do not remarkably outperform human-conducted tick drag surveys but are faster and may be useful in niche survey situations, such as surveys for tick species with potential for high aggregations, detecting invasive species, sampling unconventional surfaces like animals or shipping containers, and surveying over large areas.

## Data Availability

All data and associated metadata are available from Koser et al. (2024): https://www.sciencebase.gov/catalog/item/66f1cd96d34e0606a9dc8599 [43].

## References

[1] Eisen RJ, Kugeler KJ, Eisen L, Beard CB, Paddock CD. Tick-borne zoonoses in the United States: persistent and emerging threats to human health. ILAR J. 2017;58:319–35. 28369515 10.1093/ilar/ilx005 PMC5610605

[2] Tiffin HS, Rajotte EG, Sakamoto JM, Machtinger ET. Tick control in a connected world: challenges, solutions, and public policy from a United States border perspective. Trop Med Dis. 2022;7:388. 10.3390/tropicalmed7110388 PMC9695313 36422939

[3] Sonenshine DE. Range expansion of tick disease vectors in North America: implications for spread of tick-borne disease. Int J Env Res Pub He. 2018;15:478. 10.3390/ijerph15030478 PMC5877023 29522469

[4] Anderson K, Ezenwa VO, Jolles AE. Tick infestation patterns in free ranging African buffalo (*Syncercus caffer*): effects of host innate immunity and niche segregation among tick species. Int J Parasitol Parasit Wildl. 2013;2:1–9. 10.1016/j.ijppaw.2012.11.002. 10.1016/j.ijppaw.2012.11.002 PMC3862501 24533310

[5] Samuel WM. Factors affecting epizootics of winter ticks and mortality of moose. Alces. 2007;43:10.

[6] Tsao JI, Hamer SA, Han S, Sidge JL, Hickling GJ. The contribution of wildlife hosts to the rise of ticks and tick-borne diseases in North America. J Med Entomol. 2021;58:1565–87. 33885784 10.1093/jme/tjab047

[7] Swei A, Couper LI, Coffey LL, Kapan D, Bennett S. Patterns, drivers, and challenges of vector-borne disease emergence. Vector Borne Zoon Dis. 2020;20:159–70. 10.1089/vbz.2018.2432 PMC7640753 31800374

[8] Mansfield KL, Jizhou L, Phipps LP, Johnson N. Emerging tick-borne viruses in the 21st century. Front Cell Infect Microbiol. 2017;7:298. 28744449 10.3389/fcimb.2017.00298 PMC5504652

[9] Altizer S, Ostfeld RS, Johnson PT, Kutz S, Harvell CD. Climate change and infectious diseases: from evidence to a predictive framework. Science. 2013;341:514–9. 23908230 10.1126/science.1239401

[10] Cunze S, Glock G, Kochmann J, Klimpel S. Ticks on the move—climate change-induced range shifts of three tick species in Europe: current and future habitat suitability for *Ixodes ricinus* in comparison with *Dermacentor reticulatus* and *Dermacentor marginatus*. Parasitol Res. 2022;121:2241–52. 35641833 10.1007/s00436-022-07556-x PMC9279273

[11] Ogden NH, Lindsay LR. Effects of climate and climate change on vectors and vector-borne diseases: ticks are different. Trend Parasitol. 2016;32:646–56. 10.1016/j.pt.2016.04.015. 10.1016/j.pt.2016.04.015 27260548

[12] Ogden NH, Ben Beard C, Ginsberg HS, Tsao JI. Possible effects of climate change on ixodid ticks and the pathogens they transmit: predictions and observations. J Med Entomol. 2020;58:1536–45. 10.1093/jme/tjaa220. 10.1093/jme/tjaa220 PMC9620468 33112403

[13] Salomon J, Hamer SA, Swei A. A beginner’s guide to collecting questing hard ticks (Acari: Ixodidae): a standardized tick dragging protocol. J Insect Sci. 2020;20. 10.1093/jisesa/ieaa073. 33135760 10.1093/jisesa/ieaa073 PMC7604844

[14] Newman BC, Sutton WB, Wang Y, Schweitzer CJ, Moncayo AC, Miller BT. A standardized method for the construction of a tick drag/flag sampling approach and evaluation of sampling efficacy. Exp Appl Acarol. 2019;79:433–46. 31677026 10.1007/s10493-019-00429-6

[15] Dantas-Torres F, Lia RP, Capelli G, Otranto D. Efficiency of flagging and dragging for tick collection. Exp Appl Acarol. 2013;61:119–27. 23417703 10.1007/s10493-013-9671-0

[16] Dobson ADM. Ticks in the wrong boxes: assessing error in blanket-drag studies due to occasional sampling. Parasit Vectors. 2013;6:344. 10.1186/1756-3305-6-344. 24321224 10.1186/1756-3305-6-344 PMC4029458

[17] Kjellander PL, Aronsson M, Bergvall UA, Carrasco JL, Christensson M, Lindgren P-E, et al. Validating a common tick survey method: cloth-dragging and line transects. Exp Appl Acarol. 2021;83:131–46. 33242188 10.1007/s10493-020-00565-4 PMC7736024

[18] Holcomb KM, Khalil N, Cozens DW, Cantoni JL, Brackney DE, Linske MA, et al. Comparison of acarological risk metrics derived from active and passive surveillance and their concordance with tick-borne disease incidence. Tick Tick Borne Dis. 2023;14:102243. 10.1016/j.ttbdis.2023.102243 PMC10885130 37611506

[19] Cristescu RH, Foley E, Markula A, Jackson G, Jones D, Frère C. Accuracy and efficiency of detection dogs: a powerful new tool for koala conservation and management. Sci Rep. 2015;5:8349. 10.1038/srep08349. 25666691 10.1038/srep08349 PMC4322364

[20] Duggan JM, Heske EJ, Schooley RL, Hurt A, Whitelaw A. Comparing detection dog and livetrapping surveys for a cryptic rodent. J Wildl Manag. 2011;75:1209–17.

[21] Lee DH, Cullum JP, Anderson JL, Daugherty JL, Beckett LM, Leskey TC. Characterization of overwintering sites of the invasive brown marmorated stink bug in natural landscapes using human surveyors and detector canines. PLoS One. 2014;9:e91575. 10.1371/journal.pone.0091575. 24717734 10.1371/journal.pone.0091575 PMC3981664

[22] Thompson SA, Thompson GG, Withers PC, Bennett EM. Conservation detection dog is better than human searcher in finding bilby (*Macrotis**lagotis*) scats. Aust Zool. 2020;41:86–93. 10.7882/AZ.2020.012.

[23] Orkin JD, Yang Y, Yang C, Yu DW, Jiang X. Cost-effective scat-detection dogs: unleashing a powerful new tool for international mammalian conservation biology. Sci Rep. 2016;6:34758. 10.1038/srep34758. 27721442 10.1038/srep34758 PMC5056371

[24] Cristescu RH, Miller RL, Frère CH. Sniffing out solutions to enhance conservation: how detection dogs can maximise research and management outcomes, through the example of koalas. Aust Zool. 2020;40:416–32. 10.7882/az.2019.030.

[25] Beebe SC, Howell TJ, Bennett PC. Using scent detection dogs in conservation settings: a review of scientific literature regarding their selection. Front Vet Sci. 2016. 10.3389/fvets.2016.00096. 27840815 10.3389/fvets.2016.00096 PMC5083854

[26] Long RA, Donovan TM, Mackay P, Zielinski WJ, Buzas JS. Comparing scat detection dogs, cameras, and hair snares for surveying carnivores. J Wildl Manag. 2007;71:2018–25.

[27] Jones H, Pekins PJ, Kantar LE, O’Neil M, Ellingwood D. Fecundity and summer calf survival of moose during 3 successive years of winter tick epizootics. Alces. 2017;53:85–98.

[28] DelGiudice GD, Peterson RJ, Samuel WM. Trends of winter nutritional restriction, ticks, and numbers of Moose on Isle Royale. J Wildl Manag. 1997;61:9.

[29] Wunschmann A, Armien AG, Butler E, Schrage M, Stromberg B, Bender JB, et al. Necropsy findings in 62 opportunistically collected free-ranging moose (*Alces alces*) from Minnesota, USA (2003–13). J Wildl Dis. 2015;51:157–65. 10.7589/2014-02-037. 25390764 10.7589/2014-02-037

[30] Zarnke RL, Samuel WM, Franzmann AW, Barrett R. Factors influencing the potential establishment of the winter tick (*Dermacentor albipictus*) in Alaska. J Wildl Dis. 1990;26:412–5. 10.7589/0090-3558-26.3.412. 2388366 10.7589/0090-3558-26.3.412

[31] Chenery ES, Harms NJ, Mandrak NE, Molnár PK. First records of *Dermacentor albipictus* larvae collected by flagging in Yukon, Canada. Parasit Vectors. 2020. 10.1186/s13071-020-04425-3. 33176864 10.1186/s13071-020-04425-3 PMC7656712

[32] Leo SS, Samuel WM, Pybus MJ, Sperling FA. Origin of *Dermacentor albipictus* (Acari: Ixodidae) on elk in the Yukon, Canada. J Wildl Dis. 2014;50:544–51. 10.7589/2013-03-078. 24779459 10.7589/2013-03-078

[33] Brinton EP, Beck DE, Allred DM. Identification of the adults, nymphs and larvae of ticks of the genus *Dermacentor**Koch* (Ixodidae) in the western United States. Brigh Young Univ Sci Bull Biological Ser. 1965;5:1.

[34] Bates D, Maechler M, Bolker B, Walker S, Christensen RHB, Singmann H, et al. Package ‘lme4’. 2015;12:2. URL https://lme4.r-forge.r-project.org/.

[35] Team RC: R: a language and environment for statistical computing. R foundation for statistical computing. 2023.

[36] Wisely SM, Glass GE. Advancing the science of tick and tick-borne disease surveillance in the United States. Insects. 2019;10. 10.3390/insects10100361. 31635108 10.3390/insects10100361 PMC6835491

[37] Lippi CA, Ryan SJ, White AL, Gaff HD, Carlson CJ. Trends and opportunities in tick-borne disease geography. J Med Entomol. 2021;58:2021–9. 10.1093/jme/tjab086. 34027972 10.1093/jme/tjab086 PMC8577696

[38] Wasser SK, Davenport B, Ramage ER, Hunt KE, Parker M, Clarke C, et al. Scat detection dogs in wildlife research and management: application to grizzly and black bears in the Yellowhead Ecosystem, Alberta, Canada. Can J Zool. 2004;82:475–92.

[39] Showler AT, Pérez de León A, Saelao P. Biosurveillance and research needs involving area-wide systematic active sampling to enhance integrated cattle fever tick (Ixodida: Ixodidae) eradication. J Med Entomol. 2021;58:1601–9. 33822110 10.1093/jme/tjab051

[40] Trout Fryxell RT, Vann DN, Butler RA, Paulsen DJ, Chandler JG, Willis MP, et al. Rapid discovery and detection of *Haemaphysalis longicornis* through the use of passive surveillance and collaboration: building a state tick-surveillance network. Int J Env Res Pub He. 2021;18:7980. 10.3390/ijerph18157980 PMC8345789 34360274

[41] Thompson AT, White SA, Doub EE, Sharma P, Frierson K, Dominguez K, et al. The wild life of ticks: Using passive surveillance to determine the distribution and wildlife host range of ticks and the exotic *Haemaphysalis**longicornis*, 2010–2021. Parasit Vectors. 2022;15:1–13. 36127708 10.1186/s13071-022-05425-1 PMC9487032

[42] Chenery ES, Harms JN, Fenton H, Mandrak NE, Molnár PK. Revealing large-scale parasite ranges: an integrated spatiotemporal database and multisource analysis of the winter tick. Ecosphere. 2023;14:e4376.

[43] Koser TM, Tomaszewski EM, Cross PC, Hurt A. Working dog tick survey transect results and cost comparison in Jackson, Wyoming in Oct–Nov 2021. US Geol Surv Data Releas. 2024. 10.5066/P13A6AWF.

